# Are Public Health Organizations Tweeting to the Choir? Understanding Local Health Department Twitter Followership

**DOI:** 10.2196/jmir.2972

**Published:** 2014-02-26

**Authors:** Jenine K Harris, Bechara Choucair, Ryan C Maier, Nina Jolani, Jay M Bernhardt

**Affiliations:** ^1^Brown SchoolWashington University in St LouisSt Louis, MOUnited States; ^2^Chicago Department of Public HealthChicago, ILUnited States; ^3^Feinberg School of MedicineNorthwestern UniversityChicago, ILUnited States; ^4^Center for Public Health Systems ScienceWashington University in St LouisSt Louis, MOUnited States; ^5^National Association of County and City Health OfficialsWashington DC, DCUnited States; ^6^Center for Digital Health and WellnessCollege of Health and Human PerformanceUniversity of FloridaGainesville, FLUnited States

**Keywords:** local health department, Twitter, social media

## Abstract

**Background:**

One of the essential services provided by the US local health departments is informing and educating constituents about health. Communication with constituents about public health issues and health risks is among the standards required of local health departments for accreditation. Past research found that only 61% of local health departments met standards for informing and educating constituents, suggesting a considerable gap between current practices and best practice.

**Objective:**

Social media platforms, such as Twitter, may aid local health departments in informing and educating their constituents by reaching large numbers of people with real-time messages at relatively low cost. Little is known about the followers of local health departments on Twitter. The aim of this study was to examine characteristics of local health department Twitter followers and the relationship between local health department characteristics and follower characteristics.

**Methods:**

In 2013, we collected (using NodeXL) and analyzed a sample of 4779 Twitter followers from 59 randomly selected local health departments in the United States with Twitter accounts. We coded each Twitter follower for type (individual, organization), location, health focus, and industry (eg, media, government). Local health department characteristics were adopted from the 2010 National Association of City and County Health Officials Profile Study data.

**Results:**

Local health department Twitter accounts were followed by more organizations than individual users. Organizations tended to be health-focused, located outside the state from the local health department being followed, and from the education, government, and non-profit sectors. Individuals were likely to be local and not health-focused. Having a public information officer on staff, serving a larger population, and “tweeting” more frequently were associated with having a higher percentage of local followers.

**Conclusions:**

Social media has the potential to reach a wide and diverse audience. Understanding audience characteristics can help public health organizations use this new tool more effectively by tailoring tweet content and dissemination strategies for their audience.

## Introduction

In the United States, local health departments are governmental agencies providing essential public health services in cities, counties, metropolitan areas, districts, and tribal areas [1]. One of the services provided by local health departments throughout the United States is informing and educating constituents about health [2]. Communication with constituents about public health issues and health risks is also among the recently developed standards required of local health departments for accreditation [3]. Past research found that only 61% of local health departments met standards for informing and educating constituents [4], suggesting a considerable gap between current practices and best practice. In addition, the most recent data from the national professional organization representing local health departments, the National Association of County and City Health Officials (NACCHO), shows nearly half of local health departments (45.5%) received budget cuts in 2010 [5], indicating a growing need for local health departments to implement low-cost strategies when providing essential services in difficult economic times.

Approximately 80% of US adult Internet users have searched for health information online, making the Internet second only to health care providers as a source for health information [6]. Social media, such as Twitter, Facebook, and YouTube, have emerged as extremely popular online platforms for health information seeking and information sharing [6-8]. Social media platforms are increasingly used by health care providers [7,9-11] and public health practitioners [12-21] to find and share health information, conduct surveillance, and manage large-scale emergency situations. Twitter is a popular free-to-use social media application for microblogging, or sending and receiving brief, direct, one-to-many messages or “tweets” [12,22]. Twitter accounts can be followed by other Twitter users, allowing individuals or organizations to receive information and share or retweet these messages to others in their network. Retweeting is forwarding a tweet sent by another user, usually adding “RT” to the text to show the tweet is not original. As of 2013, approximately 18% of US adults reported using Twitter [23]. Twitter use among US adults is associated with younger age and is higher among black non-Hispanic Internet users. However, Twitter use is independent of gender, educational attainment, and income [24,25], suggesting it may provide an important new channel for disseminating public health messages to groups, such as lower socioeconomic status, households that have traditionally been more difficult to reach with health information.

Understanding the audience intentionally receiving health information is key to successful health communication [26-29]. We know the general characteristics of Twitter users overall; however, little is known about the composition of social media audiences for specific types of Twitter users, such as local health departments [24,30,31]. To aid in developing the evidence base for local health department social media use, a promising low-cost strategy for educating and informing constituents about health, we examined: (1) the general characteristics of local health department Twitter followers, such as whether they are individuals or organizations; (2) the relationship between health department characteristics, such as size and staffing, and Twitter follower characteristics; and (3) the relationship between local health department Twitter use, such as tweet frequency and Twitter follower characteristics.

## Methods

### Overview

As of July 30, 2012, we identified 217 local health departments nationwide using Twitter (identification process described elsewhere [32]) out of more than 2500 local health departments in the United States. At a minimum, Twitter users choose a username when opening an account. They may also enter a photo, their full name, location, description, and affiliated website link, although this information is not required for an account. In 2013, we used NodeXL [33], an open-source network data collection tool, to collect the Twitter followers for each of the 217 local health departments using Twitter. All 146,013 followers were consolidated into a single data file comprised of more than 98,000 unique Twitter users. To better understand the composition of this large group of followers, we constructed a representative stratified random sample of approximately 5000 Twitter users for in-depth coding. To ensure local health departments with fewer followers were represented in the dataset, we first selected a random sample of 59 of the 217 local health departments. We then compared the local health departments in the sample to those not in the sample to determine whether the sampled health departments were representative of the population of local health departments using Twitter. The comparison included 3 characteristics associated with Twitter adoption and Twitter followership for a local health department: jurisdiction population, spending per capita, and public information officer staffing [32]. We found no significant differences between those selected and those not selected for jurisdiction population size (*t*_199_=0.60; *P*=.55), spending per capita (*t*_166_=1.34; *P*=.18), and employment of a public information officer (χ^2^_1_=0.4; *P*=.51). We took a random sample of 100 followers from each of the 59 local health departments. In all, 21 of 59 (36%) local health departments had fewer than 100 followers, so these local health departments contributed all of their followers to the sample for coding, resulting in a sample of 4779 Twitter followers from 59 local health departments.

### Coding

**Coding scheme for Twitter followers (N=4779) from 59 local health departments nationwide.**

Location: Where is the Twitter follower located?

- In-state where local health department is located
- Out-of-state (in United States)
- Outside United States
- Unable to determine (in United States)
- Unable to determine

Type: Based on the username and description, does the follower account appear to be for an...?

- Individual
- Organization/business
- Unable to determine

Industry: Which of the following industries best describes the follower based on description or linked website?

- Spam
- Unable to determine
- Health-focused
  - Private user
  - Educational institution
  - US government
  - Local
  - State
  - National
  - Campaign/program
  - Not-for-profit (nongovernmental)
  - Local
  - State
  - National
  - International
  - Campaign/program
  - For profit
  - Hospital or hospital system
  - Private physician or physician offices
  - Drug company or representative
  - Managed care
  - Patient advocate
  - Medical device maker, seller
  - Fitness center/gym/personal training
  - Diet/nutrition (eg, Weight Watchers, Jenny Craig)
  - Assisted living
  - Media
  - TV
  - Newspaper/magazine (print media)
  - Radio
  - Social media or website (not affiliated with TV, radio, newspaper)
  - Public relations firm
  - Other (make note)
- Not health-focused
  - Private user
  - Educational institution
  - US government
  - Local
  - State
  - National
  - Campaign/program
  - Not-for-profit (nongovernmental)
  - Local
  - State
  - National
  - International
  - Campaign/program
  - For profit
  - Media
  - TV
  - Newspaper/magazine (print media)
  - Radio
  - Social media or website (not affiliated with TV, radio, newspaper)
  - Public relations firm
  - Other (make note)

Project team members reviewed the Twitter follower information to develop a coding scheme with 3 broad categories: location, type, and industry. Location was an indicator of whether the Twitter follower was in the same state with the local health department, out-of-state (in the United States), outside the United States, unable to tell but within the United States, and unable to determine. Type of follower included 3 categories: individual, organization/business, and unable to determine. To discern individual followers, we looked for information written in the first person using “I” or “my” or other descriptors or wording indicating the user was an individual. Organizational accounts, on the other hand, often included a statement of organizational purpose. Industry was divided into 4 subgroups: health-related, non-health-related, spam, and unable to determine. Classification of an account as spam occurred when the account did not appear to be a legitimate person or business. For example, one spam account was following 2002 others, had 67 followers, and had never tweeted. Another spam-classified account had no user description, 49 followers, and had tweeted over 1000 times with nearly all the tweets being retweeted from another user, suggesting automated retweeting by a spambot, or a program that is designed to send out spam. Within industry were several specific types of organizations. If the industry was not easy to glean from the Twitter profile (eg, “St. Mary’s is a nonprofit hospital”), we searched the user’s website and often found the type on the About Us page. Consistent with previous research [34], industry was classified for both organizations and individual users whose Twitter profiles indicated they were representing an organization. Textbox 1 includes a summary of the coding scheme.

To test the reliability of the coding scheme, 4 coders coded data from the same 100 Twitter followers (2.09%) randomly selected from the overall sample of 4779. Krippendorff’s alpha for nominal data was computed for each of the 3 broad coding categories (type, location, industry). For follower type, alpha was .70 (95% CI .63-.77), for follower location alpha was .88 (95% CI .84-.91), and for industry alpha was .68 (95% CI .64-.72). Given these acceptable reliability scores, the full dataset was divided and coded independently by the 4 coders.

### Analyses

For the first aim of the study, understanding the general characteristics of local health department Twitter followers, frequencies and percentages were used to examine the distribution of follower types. Chi-square tests were used to determine whether certain types of followers were more or less likely than expected given the overall distribution of followers. For example, were individual users more or less likely than expected to be local? Standardized residuals were calculated for significant chi-square test results; standardized residuals greater than 1.96 indicated significantly more followers than expected fell into a given category, whereas standardized residuals less than –1.96 indicated significantly fewer followers than expected fell into a category. Followers classified as “unable to determine” were omitted from analyses.

The second and third aims were to understand the relationship between local health department characteristics, Twitter use, and characteristics of their Twitter followers. For these aims, follower data was aggregated by local health department to compare the proportions of follower types in local health departments varying by (1) Twitter use (number of followers, number of tweets) and (2) resources (population size, staffing, funding per capita). Twitter usage was obtained through NodeXL in April 2013. Local health department resource information was obtained from the NACCHO 2010 Profile Study.

Because local health departments provide services to their local constituents, it is important to know what is associated with reaching local individuals. Local health departments with a public information officer may have more, and more organized, information-sharing efforts in the local community given this specialized staffing. Larger jurisdiction population and a higher number of tweets are associated with more followers [32], but it is not known whether these factors also influence the proportion of local followers. To examine what is associated with reaching local individuals, we hypothesized that:

1. Local health departments with a public information officer have a higher proportion of local followers and individual followers than local health departments without a public information officer.
2. The more Twitter followers and tweets a local health department has, the higher the percentages of local and individual Twitter followers there will be.
3. The larger the jurisdiction population, the higher the percentages of local Twitter followers and individual Twitter followers there will be.

Other ways for local health departments to reach and inform local constituents could be through local media and local government. Journalists have adopted social media as sources of information, with more than 30% of print journalists deeming social media as important or very important as of 2009 [35]. Local media following local health departments on Twitter may facilitate secondary dissemination by covering tweeted topics in local newspapers or on local radio or television broadcasts. Media advocacy is one strategy that may work to influence policy [36]; local government following local health departments on Twitter could use tweeted information to support local policy development, passage, or enforcement. To understand more about connections with local media and government, we hypothesized:

1. Local health departments with a public information officer have a higher proportion of local media followers (TV, radio, and newspaper) and local government followers than local health departments without a public information officer.
2. The larger the jurisdiction population, the higher the percentages of local media (TV, radio, and newspaper) followers and local government followers there will be.

Hypotheses 1, 3, 4, and 5 aid in addressing aim 2 (understanding the relationship between local health department characteristics and Twitter use), whereas hypothesis 2 aids in addressing aim 3 (understanding the relationship between local health department characteristics and characteristics of their Twitter followers).

## Results

### Summary

The 59 local health departments in the sample had between 9700 and 3 million constituents in their local jurisdictions according to the 2010 NACCHO Profile Study. In all, 29 (49%) of the departments reported having a public information officer, whereas 23 (39%) reported not having one (7/59, 12% were missing data on this staffing). The median number of Twitter followers was 218 (range 7-11,827; mean 770.1, SD 1688.0); the median number of sent tweets per health department since adopting Twitter was 324 (range 0-5849; mean 667.9, SD 1083.1). Health departments in the sample joined Twitter between June 2008 and January 2012; more than half (34/59, 58%) joined in 2009.

### Twitter Follower Characteristics

Overall, we found that local health departments had more Twitter followers that were organizations (2591/4434, 58.43%) than individuals (1843/4434, 41.57%). Of the 1843 individuals, 1267 (68.75%) were private personal accounts (private users not affiliated with a specific organization or business). The 1267 private individuals comprised 29.07% of the follower industry classifications for the 4359 classified by industry (Table 1). The most common type of organizational Twitter follower was from the for-profit sector (n=1053), comprising 24.16% of the 4359 followers classified by industry. More than half of followers classified as health-focused (2592/4340, 59.72%) did not include a health focus in their account information and 2149 of 3878 location-classified followers (55.42%) were in the same state as the health department they were following. A summary of the type, industry, and location of local health department Twitter followers is shown in Table 1. Users classified as “unable to determine” during coding were not included in analyses or shown in Table 1. Overall, those classified as unable to determine comprised 7.22% of the type category (345/4779), 8.79% of the industry category (420/4779), 9.19% of the health-focus category (439/4779), and 18.85% of the location category (901/4779).

Omitting the private user category because it only applied to individuals, follower industry was significantly associated with follower type (χ^2^_6_=112.6, *P*<.001). Standardized residuals indicated that there were more organizations and fewer individuals than expected in the nonprofit category (Table 1). The opposite was true for the media category with significantly more individuals than expected and fewer organizations than expected. In addition, fewer individuals than expected fell into the other category for industry. Examples of the descriptions entered by users for several common categories of local health department Twitter followers are shown in Table 2.

Considering all cases (including private users), industry was associated with health focus (χ^2^_7_=783.3, *P*<.001). The proportion of health-focused followers was higher than expected for education, government, nonprofit, and other. The proportion of health-focused followers was lower than expected in the private user, for-profit, and media categories. There was a significant association between type and health focus (χ^2^_2_=308.3, *P*<.001) with more organizations and fewer individuals being health-focused than expected. Type and location were also significantly associated (χ^2^_3_=47.0, *P*<.001), with more organizations than expected being outside the state from the local health department and more individuals than expected being within the same state as the local health department.

**Table 1:** Characteristics of local health department Twitter followers.

Characteristic		All		Individuals		Organizations		*P*
		n	%	n	%	n	%	
**Type**		4434						
	Individual	1843	41.57	—	—	—	—	
	Organization	2591	58.43	—	—	—	—	
**Industry**		4359		477		1551		<.001^a^
	Private user	1267	29.07	—	—	—	—	
	Education	160	3.67	24	5.03	136	5.33	
	Government	497	11.40	71	14.88	426	16.70	
	Nonprofit	556	12.76	31	6.50^b^	525	20.58^c^	
	For-profit	1053	24.16	174	36.48	868	34.03	
	Media	599	13.74	160	33.54^c^	422	16.54^b^	
	Other	192	4.40	16	3.35^b^	170	6.67	
	Spam	35	0.80	1	0.21	4	0.16	
**Health focus**		4340		1738		2563		<.001
	Yes	1748	40.28	419	24.11^b^	1303	50.83^c^	
	No	2592	59.72	1319	75.89^c^	1260	49.16^b^	
**Location**		3878		1371		2417		<.001
	In-state	2149	55.42	840	61.27^c^	1281	53.00^b^	
	Out-of-state	1195	30.81	363	26.48^b^	815	33.72^c^	
	In US state unknown	337	8.69	41	2.99^b^	147	6.08^b^	
	Outside US	197	5.08	127	9.26	174	7.20	

^a^Private person was omitted for the purposes of bivariate analysis.

^b^More followers than expected fell into this category (standardized residuals >1.96).

^c^Fewer followers than expected fell into this category (standardized residuals <–1.96).

**Table 2:** Examples of common categories of local health department Twitter followers.

Follower type	Health focus	Twitter user description
Private person	No	I love music and travel. Watching movies and sunsets. I like to play World of Warcraft.
	Yes	Huggable Health Educator
Nonprofit organizations	No	IBA is a nonprofit agency dedicated to empowering individuals and families through education, economic development, technology and the arts.
	Yes	Advocating for the health and dignity of Denver’s injection drug users in accordance with #harmreduction principles. Syringe exchange and Naloxone Save Lives!
Media individuals	No	San Diego reporter at The Daily Transcript. Freelance sports writer for Southwest Riverside News Network. Associate Producer for KUSI Prep Pigskin Report.
	Yes	Journalist covering medical/health & fitness and writing features for the Tyler Morning Telegraph.

### Local Health Department Characteristics

On average, more than half of a local health department’s Twitter followers were from within the state (5%, range 10%-91%). Consistent with the overall composition of the follower sample, 70% (41/59) of local health departments were followed by a higher proportion of organizations than individuals (range 22%-83% organizational followers). An even greater number of departments had a majority of non–health-focused followers (compared to health) with 44 of 59 (75%) departments having more than 50% non–health-focused followers (range 17%-86%). The average percentage of in-state media followers (TV, newspaper, radio) was 6% (SD 5.8) with a range from 0 to 25%. The average percentage of in-state government followers was 7% (SD 8.1) with a range from 0 to 43%.

### Local and Individual Followers

We hypothesized that the local health departments with a public information officer have a higher proportion of local followers and individual followers than local health departments without a public information officer. A *t* test indicated that local health departments with a public information officer did have a significantly higher percentage of local followers (*t*_50_=2.3, *P*=.03) than local health departments without a public information officer. Local health departments with a public information officer had an average of 63.9% (range 15.1%-90.8%) in-state followers compared to 49.3% (range 9.5%-90.9%) for those without a public information specialist. However, another *t* test (*t*_35.9_=–1.71; *P*=.10) found no significant difference in the proportion of followers that were individuals among local health departments with a public information officer (mean 44.1%, range 28.6%-55.9%) compared to those without a public information officer (mean 38.9%, range 21.7%-66.7%).

We hypothesized that, the more Twitter followers and tweets a local health department had, the higher the percentages of local and individual Twitter followers there was. There was a strong positive correlation between the number of followers and the number of tweets for a local health department (*r*=0.74, *P*=.049), so we tested only the relationship between number of tweets and follower characteristics. We chose number of tweets because it is a characteristic the local health department can modify as part of a social media strategy, unlike the number of followers. We found a weak, positive, and statistically significant association between the number of tweets a health department had sent and the proportion of its followers classified as individuals (*r*=.32, *P*=.02); however, there was no significant association between number of tweets and the proportion of in-state followers.

We hypothesized that, the larger the jurisdiction population, the higher the percentages of local Twitter followers and individual Twitter followers. A correlation coefficient indicated a positive and significant, although weak, association between jurisdiction population and percent of followers who were local (*r*=0.33, *P*=.01). However, population size had no significant association with proportion of followers who were individuals.

### Local Media and Local Government Followers

We also hypothesized that local health departments with a public information officer have a higher proportion of local media followers (TV, radio, and newspaper) and local government followers than local health departments without a public information officer. The *t* tests demonstrated no significant association between having a public information officer and having more local media or local government followers.

Finally, we hypothesized that, the larger the jurisdiction population, the higher the percentages of local media (TV, radio, and newspaper) followers and local government followers. The size of the jurisdiction population was not associated with the percent of followers who were local government or local media.

## Discussion

### Principal Findings

The results of our research reveal that local health departments on Twitter in the United States are followed by more organizations than individuals. Many of the organizations are health-focused, out-of-state, and from the education, government, and nonprofit sectors, suggesting that there may be a communication network comprised of organizations with a health-related mission developing on Twitter. Individual followers of local health departments, on the other hand, tended to be locally based and largely did not have a health focus, indicating that, at least where individuals are concerned, local health departments may not be just tweeting to members of the public who are already health-focused (ie, the choir).

Evidence of a network of public health organizations developing on Twitter is consistent with at least 2 recent studies [37,38]. The first study demonstrated that many of the local health departments on Twitter follow the same set of government agencies, media, nonprofit organizations, professional associations, educational institutions, and for-profit organizations, many of which are health-focused [38]. Several organizations were followed by 100 or more local health departments, including 4 Centers for Disease Control accounts (@CDCemergency, @CDCFlu, @CDC_eHealth, @CDCgov), 2 other government agencies (@FDArecalls, @HHSGov), and 2 professional organizations (@PublicHealth, @NACCHOalerts). The second study identified a 1516 Twitter follower relationship among 182 local health departments on Twitter. Contrary to the current study, however, the follower connections among these 182 local health departments were more likely to be between organizations in the same state than to be across states [37].

Consistent with other studies [12,32], more tweeting by local health departments was associated with having more followers and, in this case, with having more individual followers (as opposed to organizational). A recent analysis of local health department tweet content indicated that local health departments are largely tweeting about healthy behaviors, most likely with the purpose of reaching individual constituents [20]. Having more individual followers appears consistent with sending these individually focused tweets. However, because more than half the Twitter audience is comprised of organizations, and growing evidence around interorganizational connections for public health on Twitter, the tweet content study [20] suggests a possible disconnect with individually focused tweets reaching a Twitter audience comprised of organizations.

Although tweeting more often and serving larger populations has been associated with having more followers overall for local health departments [39], we found these characteristics were not associated with having more local followers in general or more local media and local government followers specifically. Past research has also demonstrated that employment of a public information officer in a local health department is associated with adopting social media earlier, having more followers [39], and tweeting about specific public health topics [40]. Although local followership was significantly higher for health departments employing a public information officer in this study, having a public information officer was not associated with having more local media or government followers. Given the emerging evidence about social media activity in local health departments employing public information specialists, additional research is needed into the social media strategies of these specialized staff members and other characteristics of their health departments that might influence social media activity.

Our findings may be useful for local health departments in at least two ways. First, we identified the characteristics (eg, jurisdiction population size, employing a public information officer) and practices (eg, tweet frequency) associated with local health department Twitter follower characteristics. This information could inform strategic planning for local health departments using or considering using Twitter. For example, if a goal of a local health department is to reach greater numbers of individuals rather than organizations, their Twitter strategy could include a regular daily or weekly tweeting schedule.

Second, understanding who the Twitter followers are could help local health departments better target tweets to diverse audiences. For example, local media and policymakers may be important followers for a local health department. Standard strategies (eg, tweeting more, developing an easy-to-find user profile [41]) for increasing the number of followers may bring in more media and policymakers. However, additional research into how media and policymakers on Twitter select specific information sources may help to identify strategies for local health departments in increasing the presence of these followers. As another example, out-of-state organizations are less likely to be interested in tweets focused at the individual, as well as locally focused or locally relevant tweets about health and health-related events. However, these followers may be interested in learning about innovative or successful local health department programs and best practices. Local health departments wishing to connect and engage more with this existing audience might program their Twitter accounts as dissemination platforms to reach these organizations with relevant information about successful strategies and programs.

**Figure 1:**
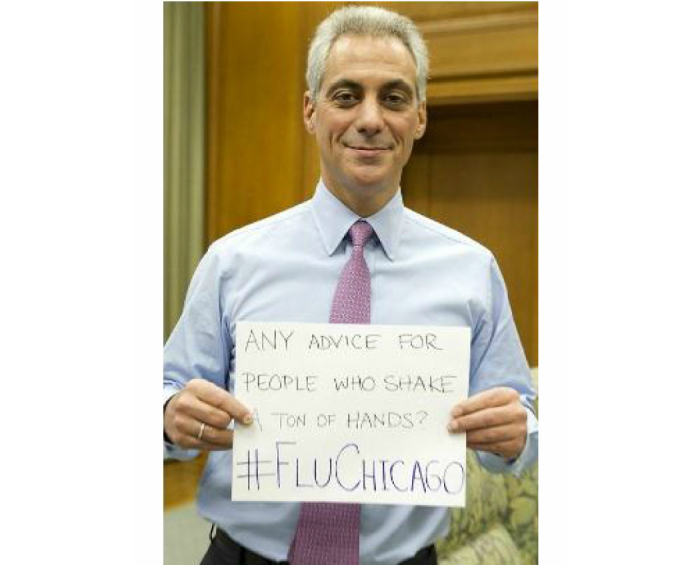
Chicago mayor Rahm Emanual participating in the #FluChicago 2013 Twitter chat about flu prevention with @ChiPublicHealth during flu season (Photo from Chicago Mayor’s Office, @ChicagosMayor [43]).

Some local health departments are already focused on reaching specific individual and organizational audiences. For example, the Chicago Department of Public Health has begun conducting campaigns using social media as a dissemination channel and making explicit efforts to interact with local individuals through Twitter activities, such as Twitter live chats. One of these events took place in early 2013, when Dr Julie Morita of the Chicago Department of Public Health answered questions from Chicagoans in a Twitter live chat about flu (using hashtag #FluChicago) just as local news coverage of flu was increasing. Through their official Twitter account (@ChiPublicHealth), she answered questions ranging from the Chicago mayor asking about prevention when in close contact with many people (Figure 1) to constituents asking about the severity of the flu season, the safety and location of flu shots, and how long to wait before returning to work after having the flu. Although @ChiPublicHealth chose to use their Twitter feed to combat an emerging local public health problem, a recent study found examining diabetes rates and tweeting about diabetes found no association between local rates and local health department tweeting [40], suggesting that opportunities exist to increase Twitter use to address locally relevant health issues.

Other health departments have adopted Twitter with the purpose of sharing information with public health organizations. For example, local health departments across Utah made a statewide effort to adopt and use social media. A local health department practitioner at Bear River Health (@BearRiverHealth) in Utah described this strategy as follows, “Not only does it allow us an opportunity to share information, it allows us to communicate in a new way with the communities that we serve together as a state. For example, when we launch an immunization campaign we now have the ability to share the same message seamlessly across our entire state through Twitter and Facebook. We share one another’s posts, comment on status, and generally connect” (Jill Parker, personal communication, November 2012). This coordinated effort and active use of Twitter has resulted in Utah communities such as the jurisdiction of Bear River Health with a 2010 population of 163,836 to reach more than 3200 followers, more than 5 times the average number of followers for a local health department [32]. Given these innovative and varied uses of Twitter by local health departments and the composition of follower types for local health departments, future studies may wish to focus on whether and how local health departments are choosing their social media strategies and which strategies are effective at influencing follower behavior for different types of followers.

### Limitations and Conclusions

Limitations to this study include cross-sectional data, reliance on self-reported information, and a lack of information on follower engagement. For example, because many Twitter users do not include geographically specific information about their location in the user profile, the coding of local was limited to identifying whether a follower was in the same state as the local health department. Likewise, a Twitter user may have had a health focus, but did not include health-related language in their profile and was coded as nonhealth. In addition, without additional information about Twitter follower engagement, such as mentions and retweets, it is impossible to know the extent to which the followers were actively engaged with the health departments through their Twitter accounts [42]. Despite its limitations, this study provides an important first look at the characteristics of Twitter users connected to local health departments.

## Abbreviations

NACCHO: National Association of County and City Health Officials

## References

[1] (2005). National Association of County and City Health Officials.

[2] (2010). Centers for Disease Control and Prevention, National Public Health Performance Standards Program (NPHPSP).

[3] (2011). Public Health Accreditation Board Standards and Measures.

[4] Mays GP, McHugh MC, Shim K, Lenaway D, Halverson PK, Moonesinghe R, Honoré P (2004). Getting what you pay for: public health spending and the performance of essential public health services. J Public Health Manag Pract.

[5] (2011). 2010 National Profile of Local Health Departments.

[6] Fox S (2011). The social life of health information, 2011.

[7] Moorhead SA, Hazlett DE, Harrison L, Carroll JK, Irwin A, Hoving C (2013). A new dimension of health care: systematic review of the uses, benefits, and limitations of social media for health communication. J Med Internet Res.

[8] Scanfeld D, Scanfeld V, Larson EL (2010). Dissemination of health information through social networks: twitter and antibiotics. Am J Infect Control.

[9] Hawn C (2009). Take two aspirin and tweet me in the morning: how Twitter, Facebook, and other social media are reshaping health care. Health Aff (Millwood).

[10] Antheunis ML, Tates K, Nieboer TE (2013). Patients' and health professionals' use of social media in health care: motives, barriers and expectations. Patient Educ Couns.

[11] Kostkova P, Fowler D, Wiseman S, Weinberg JR (2013). Major infection events over 5 years: how is media coverage influencing online information needs of health care professionals and the public?. J Med Internet Res.

[12] Schein R, Kumanan W, Keelan J (2011). Literature review on effectiveness of the use of social media: a report for Peel public health.

[13] Jones B (2011). Mixed uptake of social media among public health specialists. Bull World Health Organ.

[14] Sublet V, Spring C, Howard J, National Institute for Occupational SafetyHealth (2011). Does social media improve communication? Evaluating the NIOSH science blog. Am J Ind Med.

[15] Signorini A, Segre AM, Polgreen PM (2011). The use of Twitter to track levels of disease activity and public concern in the U.S. during the influenza A H1N1 pandemic. PLoS One.

[16] Hagar C (2013). Crisis informatics: Perspectives of trust – is social media a mixed blessing?. Student Research Journal.

[17] Yates D, Paquette S (2011). Emergency knowledge management and social media technologies: A case study of the 2010 Haitian earthquake. International Journal of Information Management.

[18] Hingle M, Yoon D, Fowler J, Kobourov S, Schneider ML, Falk D, Burd R (2013). Collection and visualization of dietary behavior and reasons for eating using Twitter. J Med Internet Res.

[19] Chew C, Eysenbach G (2010). Pandemics in the age of Twitter: content analysis of Tweets during the 2009 H1N1 outbreak. PLoS One.

[20] Neiger BL, Thackeray R, Burton SH, Thackeray CR, Reese JH (2013). Use of twitter among local health departments: an analysis of information sharing, engagement, and action. J Med Internet Res.

[21] Thackeray R, Neiger BL, Smith AK, Van Wagenen SB (2012). Adoption and use of social media among public health departments. BMC Public Health.

[22] (2011). The health communicator's social media toolkit.

[23] Brenner J, Smith A (2013). 72% of online adults are social networking site users.

[24] Smith A, Brenner J (2012). Twitter use 2012.

[25] Eng TR, Maxfield A, Patrick K, Deering MJ, Ratzan SC, Gustafson DH (1998). Access to health information and support: a public highway or a private road?. JAMA.

[26] Lasswell H, Bryson L (1948). The structure and function of communication in society. The Communication of Ideas.

[27] Kreuter MW, McClure SM (2004). The role of culture in health communication. Annu Rev Public Health.

[28] Kreuter MW, Wray RJ (2003). Tailored and targeted health communication: strategies for enhancing information relevance. Am J Health Behav.

[29] McGuire WJ, Rice RE, Atkin CK (1989). Theoretical foundations of campaigns. Public Communication Campaigns, Second Edition.

[30] Chou WY, Hunt YM, Beckjord EB, Moser RP, Hesse BW (2009). Social media use in the United States: implications for health communication. J Med Internet Res.

[31] Duggan M, Brenner J (2013). The demographics of social media users — 2012.

[32] Harris JK, Mueller NL, Snider D (2013). Social media adoption in local health departments nationwide. Am J Public Health.

[33] Smith M, Milic-Frayling N, Shneiderman B (2010). CodePlex.

[34] Wu SS, Hofman JM, Mason WA (2011). Who says what to whom on Twitter.

[35] Lariscy RW, Avery EJ, Sweetser KD (2009). An examination of the role of online social media in journalists. Public Relations Review.

[36] Cohen L, Chavez V, Chehimi S (2010). Using media advocacy to influence policy. Prevention is Primary: Strategies for Community Well-being.

[37] Harris JK, Brownson RC, Bell RA, Maier R, Cohen E, Mueller N (2014). Twitter connections among local health departments as potential pathways for dissemination.

[38] Harris JK, Cohen EL, Brownson RC (2013). Examining the network of local health departments and their information sources on Twitter.

[39] Harris JK, Snider D, Mueller N (2013). Social media adoption and use in health departments nationwide: the state of the states. Frontiers in Public Health Services and Systems Research.

[40] Harris JK, Mueller NL, Snider D, Haire-Joshu D (2013). Local health department use of twitter to disseminate diabetes information, United States. Prev Chronic Dis.

[41] Harris JK, Maier RC, Jolani N (2013). What's in a username? Finding local health departments on Twitter. Frontiers in Public Health Services and Systems Research.

[42] Neiger BL, Thackeray R, Burton SH, Giraud-Carrier CG, Fagen MC (2013). Evaluating social media's capacity to develop engaged audiences in health promotion settings: use of Twitter metrics as a case study. Health Promot Pract.

[43] (2013). Twitter.

